# Botanical Provenance of Traditional Medicines From Carpathian Mountains at the Ukrainian-Polish Border

**DOI:** 10.3389/fphar.2018.00295

**Published:** 2018-04-05

**Authors:** Weronika Kozlowska, Charles Wagner, Erin M. Moore, Adam Matkowski, Slavko Komarnytsky

**Affiliations:** ^1^Department of Pharmaceutical Biology, Medical University of Wroclaw, Wroclaw, Poland; ^2^Plants for Human Health Institute, North Carolina State University, Kannapolis, NC, United States; ^3^Department of Plant and Microbial Biology, North Carolina State University, Raleigh, NC, United States; ^4^Department of Biology, Catawba College, Salisbury, NC, United States; ^5^Department of Food, Bioprocessing & Nutrition Sciences, North Carolina State University, Raleigh, NC, United States

**Keywords:** ethnobotany, traditional knowledge, herbal texts, bioactive compounds, functional foods, folk remedies

## Abstract

Plants were an essential part of foraging for food and health, and for centuries remained the only medicines available to people from the remote mountain regions. Their correct botanical provenance is an essential basis for understanding the ethnic cultures, as well as for chemical identification of the novel bioactive molecules with therapeutic effects. This work describes the use of herbal medicines in the Beskid mountain ranges located south of Krakow and Lviv, two influential medieval centers of apothecary tradition in the region. Local botanical remedies shared by Boyko, Lemko, and Gorale ethnic groups were a part of the medieval European system of medicine, used according to their Dioscoridean and Galenic qualities. Within the context of ethnic plant medicine and botanical classification, this review identified strong preferences for local use of St John's-wort (*Hypericum perforatum* L.), wormwood (*Artemisia absinthium* L.), garlic (*Allium sativum* L.), gentian (*Gentiana lutea* L.), lovage (*Levisticum officinale* W.D.J. Koch), and lesser periwinkle (*Vinca minor* L.). While Ukrainian ethnic groups favored the use of guilder-rose (*Viburnum opulus* L.) and yarrow (*Achillea millefolium* L.), Polish inhabitants especially valued angelica (*Angelica archangelica* L.) and carline thistle (*Carlina acaulis* L.). The region also holds a strong potential for collection, cultivation, and manufacture of medicinal plants and plant-based natural specialty ingredients for the food, health and cosmetic industries, in part due to high degree of biodiversity and ecological preservation. Many of these products, including whole food nutritional supplements, will soon complement conventional medicines in prevention and treatment of diseases, while adding value to agriculture and local economies.

## Introduction

**Figure 1:**
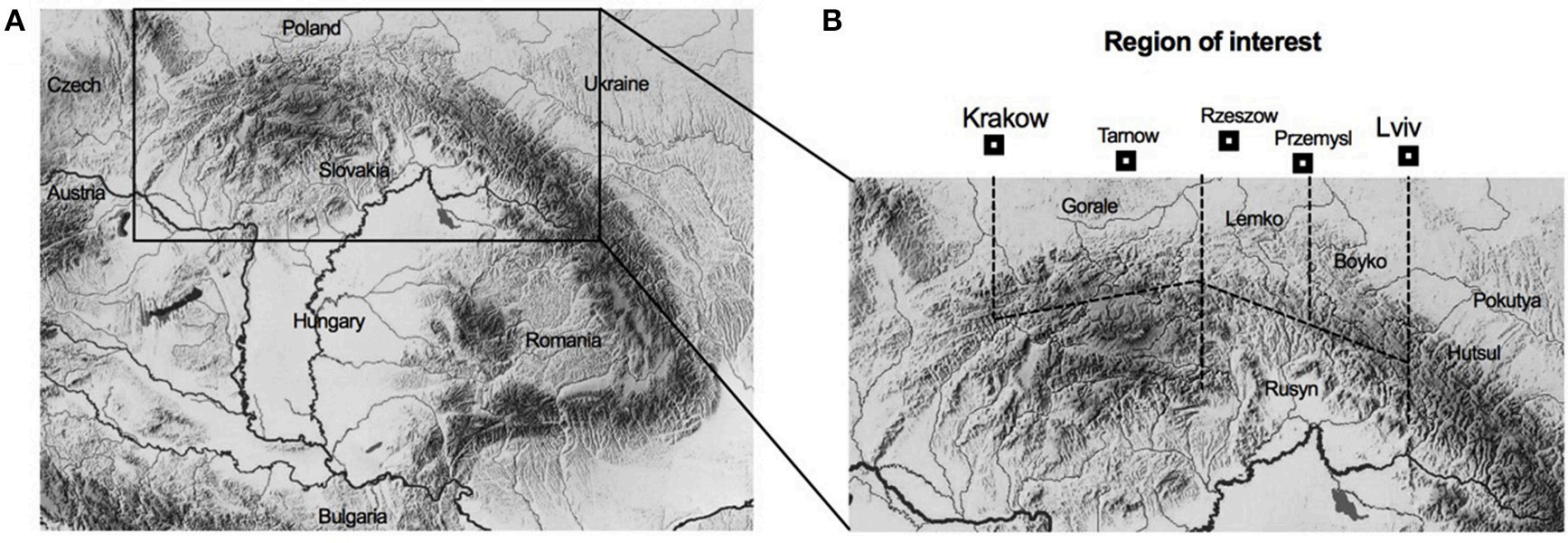
Study area that includes Beskin ridges of the Carpathin Mountains: **(A)** Schematic map of the mountain region that serves as a border between Ukraine, Poland, Slovakia, Hungary, and Romania, and **(B)** Schematic mountain areas inhibited by Gorale, Lemko, Boyko, Rusyn, and Hutsul ethinc groups.

The terms Beskids or Beskid Mountains (*Beschad Alpes Poloniae*) denote the Carpathian mountain ranges that historically separated kingdoms of Poland from Ruthenia (Ukraine) and Hungary as described in the 1269 Hungarian deed (Papee, 1891). The Beskids are approximately 600 km in length stretching from Moravian Gate in the west (Czech Republic) to Vyshkovsky Pass in the east (Ukraine) along the modern Polish-Slovak border, but are rather narrow (50–70 km in width). Gorale, Lemko, Boyko, and Rusyn ethnic groups of mixed Slavic, Vlah (Aromanian) and Thraco-Celtic origins primarily populate these areas (Figure 1). During tenth–fourteenth centuries, Beskids formed a transborder region of limited political and economic affiliation, defined by a line of defensive border settlements (La: *ultra indagines*, Ua: *zasiky*, Pl: *bron*) advancing slowly toward the mountain ridges along the Spis-Borzhava axis in the south and Myslenice-Sanok-Dolyna axis in the north (Halaga, 1961). Turka on the route to Uzhok Pass and Wietrzno-Bobrka on the route to Dukla Pass were typical examples of such defensive hillforts (Ua: *gorodysche*, Pl: *grodzisko*). In the fourteenth century, continuous colonization of the Beskids was described as of *Ruthenus, Ruthenus-Valachus, Ruthenos seu Valachos*, or *Valachus* origin (Hoshko, 1983). The ongoing debate whether these terms identified a proper ethnic affiliation of the migrants or their rustic (agricultural) vs. valachian (pastoral) background has not been settled (Stavrovsky, 1967). The transborder status, however, combined with severe geographical and socioeconomic isolation, resulted in self-treatment as a primary choice of both medicinal and spiritual healing. Even at the beginning of the twentieth century, one medical doctor served on average 9,000–17,000 inhabitants (Otchet, 1915), and a doctor's reputation and recognition was well below that of a local pharmacist or a traditional healer.

The use of plants and their preparations in European medicine, drawing upon the traditions of both the classical and Arab civilizations, was formalized by works of Hippocrates of Kos (*Corpus Hippocraticum*), Dioscorides (*De Materia Medica*), Pliny (*Naturalis Historiae*), Avicenna (*Liber Canonis*), and Galen of Pergamon (*Hidden Drugs*, survived only in Arabic translation as *Al-Adwiya al-Maktuma*) as the science of using fresh plants and dry herbs to improve human health (Cruse, 1999). Prior to 900–1200 AD, however, no written records described traditional herbal treatments used by Celtic, Slavic, and Germanic tribes that bordered the Roman Empire. First medieval manuscripts that partially documented the use of herbs in these areas were largely translations from earlier Latin works that incorporated local variations in methods of collection, preparation, and use (Singer, 1927), including *Bald's Leechbook* and *Old English* herbals (Anglo-Saxon, tenth–eleventh century), *Regimen Sanitatis* (Italian, tenth century), *Physicians of Myddvai* (Welsh, fourteenth century), and various editions of *[H]ortus Sanitatis* (1491) based on the German *Herbarius Maguntie impressus* of 1484 and *Herbarius zu Teutsch* of 1485. The latter monographs were intended to treat of cheap and homely remedies for the use of the poor, as most herbs listed in these books were native or garden plants, while very few exotic remedies were described in details.

A glimpse of medieval Slavic herbal tradition could be found in the tenth–eleventh century manuscripts *Izborniki* (Anthologies, transcribed in 1073 and 1076 for Prince Svyatoslav of Kyiv from manuscripts that had belonged to Tsar Simeon of Bulgaria) that described medicinal uses of several plants, including henbane (*Hyoscyamus niger*), hemlock (*Conium maculatum*), and wormwood (*Artemisia absinthium*). Subsequent herbal manuscripts (Ua: *travnyk, zilejnyk, vertograd*, Pl: z*ielnik*) from sixteenth to eighteenth centuries often combined traditional herbal medicines with irrational magical applications. For example, while the seventeenth century *vertograd* described management of fever and pain with cabbage (*Brassica oleracea*) and beet (*Beta vulgaris*) leaves, and infection diseases with onion (*Allium cepa*) and garlic (*Allium sativum*), it also prescribed a mixture of horse and goat manure to cure alopecia in 3 days (Krylov, 1985). Nonetheless, these manuscripts served during the late Middle Ages as veritable manuals of practical herbal medicine. Their text and images were usually mechanically transcribed from earlier works of the most varied provenance, while the successive owners often enriched the contents with personal observations and experiences.

## Oldest herbals printed in the carpathian foreland

Foundation of Prague University in 1348 and Krakow University in 1364 provided diverse educational opportunities for wealthy local families of Polish and Ruthenian descent, and led to the expansion of local medicinal and apothecary tradition. Krakow, and later Lviv, quickly developed into two influential medieval centers of apothecary, with former also serving as an important publishing site of printed herbal texts.

Here, six major monographs describing the use of medicinal plants were published between 1532 and 1613 (Table 1). The first one was a Latin reprint of *De Herbarum Virtutibus* (Naples, 1477) with plants names translated into Polish for the first time by **Simon de Lowicz** in 1532. Its significance lies in the fact that it was the first document of such length to indicate a renewed interest on this subject and to reveal direct influence of Dioscorides and Pliny. Even though there was no evidence of experimentation, and whether or not the author actually ever attempted to use any of the herbs of which he wrote, this herbal had a tremendous influence on medical and botanical literature from the early Middle Ages (Flood, 1976). One testimonial to the wider influence Macer had on subsequent medical and botanical commentators was a large number of translations of the herbal into vernacular languages and dialects. Its popularity was likely partially due to the selection of predominantly native European plants (39) or medicinal herbs cultivated in the gardens (25) over the exotic plants (13) that were difficult to procure.

**Table 1:** Medieval herbals printed in the Carpathian foothills in the fifteenth–seventeenth centuries.

**Front page**	** 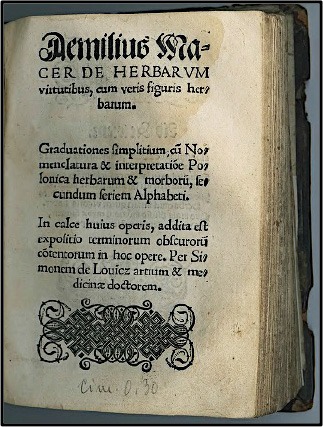 **	** 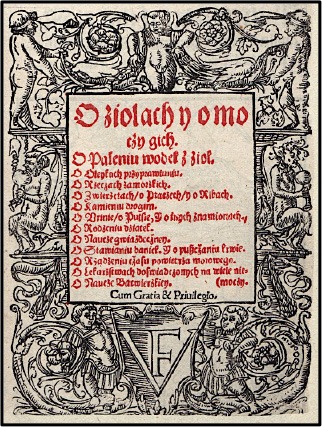 **	** 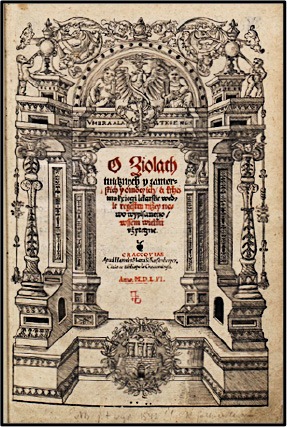 **	** 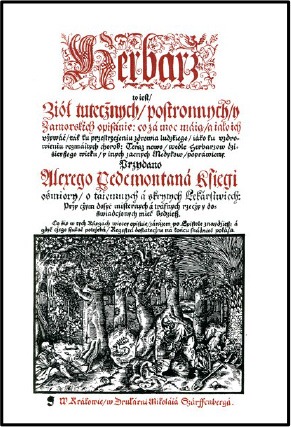 **	** 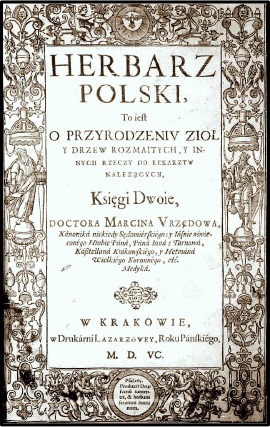 **	** 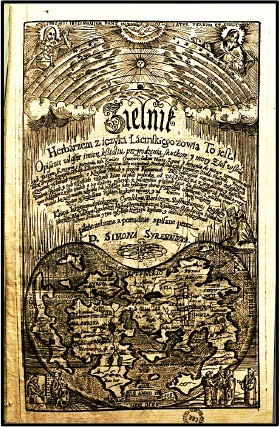 **
Author	Simon de Lowicz (-1538)	Stefan Falimirz (-1550)	Hieronymus Spiczynski (-1550)	Marcin Siennik (-1588)	Marcin de Urzedow (-1573)	Simon Syrenius (-1611)
Title	*Aemilius Macer, De herbarum virtutibus* (Krakow, 1532)	*Hortus sanitatis, O ziolach i o mocy ich* (Krakow, 1534)	*O ziolach tutecznych i zamorskich i o mocy ich* (Krakow, 1542)	*Herbarz to jest zioł tutecznych, postronnych i zamorskich opisanie…* (Krakow, 1568)	*Herbarz polski, to jest o przyrodzeniu ziol i dzew rozmaitych…* (Krakow, 1595)	*Zielnik herbarzem z iezyka lacinskiego zowia…* (Krakow, 1613)
Notes	2,756 verse lines 77 plants 13 exotic Added Polish names	433 pages 329 plants 69 exotic Translated to Polish	Adapted from Falimirz Sorted by Polish names	Adapted from Falimirz Added German names	488 pages 477 plants total 105 exotic New compilation	1,540 pages 765 plants total

**Stefan Falimirz** (Falimir, a Ruthenian courtier to Jan Tenczynski, the Duke of Podola in Krasnik) compiled various Latin *[H]ortus Sanitatis* manuscripts, translated them into Polish, and released a first popular encyclopedia of traditional medicines entitled *O ziolach i o mocy ich* (On herbs and their power) in Krakow (1534). The subjects covered herbs and herbal medicines (including the first use of Polish term *wodka* meaning “little water” to describe high proof spirit distilled from grain), medicinal properties of animals and minerals, obstetrics, surgery, and were profusely illustrated with over 550 mostly original woodcuts. Plants were described in a set of 261 articles on European and 69 exotic herbal remedies. Descriptions of plants and their use, however, were mostly translations from the previous sources and likely unknown to the author (Elbanowski, 2014). This became very clear when compared to other chapters describing animals, where Stefan provided extensive and original details on local fishes, a subject apparently of great familiarity to the author. Specifically, Stefan stated, “*Because we live in Poland, there is a need to know characteristics and use of Polish fishes, as we use them regularly. The ones that help, the ones that harm, the good, the better, and the worst*.” This statement for the first time indicated the emerging perception of inadequacy of contemporary Latin manuscripts that lacked local knowledge and tradition.

**Hieronim Spiczynski** of Wielun (a wealthy Polish councilman in Krakow) attempted an adaptation of Stefan Falimirz's work by publishing *O ziolach tutecznych y zamorskich y o mocy ich* (On local and overseas herbs and their power, 1542) with improved woodcuts. The monograph contained similar and often identical descriptions of several medical guides to practical use of herbal remedies. The catalog of herbs was focused on local plants listed under Polish and Latin names, with a shorter list of exotic plants identified only in Latin. Compared to other herbals, it contained narrower descriptions of plant species with no mention of their origin or occurrence.

**Marcin Siennik** (Merten Heüwrecher, a wealthy merchant of German origin in Krakow) compiled another adaptation of Falimirz's herbal 26 years later as *Herbarz to jest ziol tutecznych, postronnych i zamorskich opisanie* (Herbal as description of local, foreign and overseas plants, 1568). Quick comparison among these editions revealed minor changes in the order of the chapters, woodcuts, and plant names. Siennik held neither a professional title nor a degree, and seemingly edited books solely to make income in accordance with publisher's instructions. The only freedom that he enjoyed was expanding the list of plant nicknames, to which he contributed many new names, however both descriptions and uses of various remedies were transcribed intact, together with all previous errors and omissions. The resulting monograph was published under a new name, with modified woodcuts and an expanded index including German names (Rostafinski, 1888).

**Marcin de Urzedow** (a Polish Catholic priest born in Urzedow near Lublin, and a graduate of Krakow University) compiled *Herbarz polski, to jest o przyrodzeniu ziol i dzew rozmaitych* (Polish Herbal, 1595) that was printed after his death and included descriptions of 372 local plant remedies and 105 exotic herbs. Plants were listed in Latin alphabetical order and were heavily influenced by independent translations from Dioscorides *Materia medica* and Fuchs *Cometraii insignes*. Among those, only 259 chapters described wild native plants characteristic of the Polish countryside. The woodcuts, however, were the same ones used in Siennik herbal to depict 240 local herbs–and for this reason, the rest of the herbal remedies were illustrated by woodcuts that were repeated or omitted altogether. The individual reports lacked a particular order of presentation, with some chapters organized by location, description and use, while others discussed herbal properties prior to their looks and origins (Rostafinski, 1888).

**Szymon Syrenius** (Syreniusz, Syrenski) of Oswiencim, a graduate of Krakow and Padua Universities, moved to Lviv and established his medical office there in 1578. He spent next 30 years collecting botanical flora from the northern slopes of Beskids mountain ranges, including trips to more distant regions of Podolia and Pokuttya. Here he compiled the most extensive Polish herbal to date–*Zielnik herbarzem z iezyka lacinskiego zowia* (Polish herbal, 1613), printed 2 years subsequent to his death. Similarly to previously described monographs, this volume was largely based on translations of Dioscorides *Materia medica* and its adaptation by Petri Matthioli, known as *Senensis midici, commentarii in sex libros* (1565), including the original woodcuts from Matthioli's work (Rostanski, 1997). The volume provided detailed records of 765 plants, mostly from Southern and Central Europe. The information was presented in a systematic way, including plant description, area of distribution, collection and processing techniques, and medicinal properties, followed by an index of Latin, Polish, and German names.

Finally, a fairly unknown **Old-Eastern-Yiddish remedy book** (*refue-bukh*) with the Hebrew title of *Seyfer derekh ets ha-khayim* was printed in Poland in 1613. While the exact printing place of the book is unknown, as is the name of the author, Krakow was one of the possible places of its origin (Geller, 2009). Apart from a richly decorated title page, the book contains no illustrations, so technically it was not an herbal but rather a self-help botanical guide. It described 87 remedies of plant, animal, and mineral origin, with many Polish words used to identify plant names. Most botanical terms and folk remedies were referenced to the tradition of Hippocrates, Dioscorides, and Avicenna, therefore it is not clear whether this book was an original compilation of the classical sources, or a more recent adaptation of one of the herbals similar to those listed above. To this point, we would like to highlight the unknown author's experience at the Padua medical school to which he refers in the beginning of his book (Geller, 2009)–a striking parallel to the educational background of his contemporaries Marcin de Urzedow and Szymon Syrenius. This may suggest a Padua-Krakow axis as a primary route for introduction and adaptation of antique botanical knowledge into the Carpathian region. Indeed, over the course of the sixteenth century, approximately 1,400 Polish and Ruthenian students, including nearly all physicians to the Polish royal family, attended Padua University (Theodorescu, 1989). Jan Zamoyski, Polsih Crown Chancellor and graduate of Padua University, founded Zamojski Academy in 1594 and shortly after that, a Jesuit Collegium was founded in Lviv (1608), which later became Lviv Academy (1661). Together with Krakow University, these institutions rapidly became the major centers of intellectual and spiritual life in the region.

## On relevance of printed herbals to traditional botanical knowledge

The sixteenth century scholars discovered a critical obstacle in sorting and presenting botanical knowledge that had accumulated over the centuries in antique natural and medical literature, and had been translated to various languages on multiple independent occasions. The same plants were often given different names based on the place of their origin. To make things worse, the ancient texts and names of old herbal remedies were often distorted due to evolution of the language and inadequate translation or transcribing, especially when detailed descriptions of target plants were absent from the original texts. This notion did not escape the attention of herbal editors, as stated by Marcin de Urzedow in the introduction to his work: “*We are no longer satisfied by knowledge of garden and field plants, but search for new ones in the wild, in forests, on mountains and cliffs, and even places where mountain goats reached seldom*.” For this particular reason, it was very difficult to define and isolate local traditional knowledge in these monographs, as medicinal use of many ancient herbs was fully adopted and integrated into local folk knowledge. There was, however, a speculative evidence of an opposite flow of knowledge about medicinal plants from Slavic into Greco-Roman tradition: Slavic knowledge of medicinal plants supported the hypothesis of Tomaschek that the majority of so-called Dacian synonyms interpolated into the Dioscorides text from the Viennese codex are of Slavic origin (Grmek, 1959).

## Pharmacopeias that regulated apothecary tradition in the region (fifteenth–nineteenth centuries)

The official Polish and Polish-Latin scientific literature concerned with herbal medicines became comparatively extensive between fifteenth and seventeenth centuries. The first place among earlier manuscripts from the region belongs to the volume entitled *Antidotarium seu Vocabularium medicum, passim cum nominibus herbarum Germanis et Polonis rubro adscriptis* (1419), currently kept at the Jagiellonian Library in Krakow. It was likely written by an unknown physician or monk of German origin who worked in Poland and had first hand knowledge of Polish plants (Magowska, 2004). This pharmacological work was a direct adaptation from *Antidotarium parvum*, a pharmacopeia-like manuscript written by an unknown author in Salerno between eleventh and thirteenth centuries. Different editions of this work included 110–170 recipes from antiquity and early medieval writers, which were annotated with German and Polish names. We also cannot rule out the possibility that some chapters were borrowed from *Antidotarium magnum*, another work of unclear origin from the twelfth century, which may have been initiated by Constantinus Africanus and contained 1,200 recipes (Prioreschi, 2003).

**Jan Stanko** (Johannes Stanconis) of Luban near Wroclaw, a graduate of Padua University and a royal physician to Polish King Kazimierz Jagiellonczyk (Lisowski, 2007), compiled a botanical and medical glossary entitled *Incipit Antibolomenum Benedicti Parthi* in 1472 (now kept at the Archives of the Krakow Cathedral Chapter). He listed 20,000 scientific and medicinal terms of Latin, Greek, Arabic, Italian and other origins, translated into German, Polish, and Czech languages. The *Antibolomenum* served as an apothecary index identifying 433 domestic and 90 foreign plants, 219 domestic species of animals and the minerals used as the ingredients of the apothecary remedies. While useful for quick identification of medicinal ingredients and their equivalents, the manuscript was never published, and had no major impact on the development of the natural sciences at the time.

The first printed pharmacopeia that had significant impact on the apothecary tradition of Poland was compiled by a German physician **Johann Placotomus** (Breitschneider) under the title of *Pharmacopoeia in compendium redacta* and published in 1560. This book was developed on the basis of *Dispensatorium* written by Valerius Cordus (1546). The work included information on individual *materia medica* (*simples*) and complex recipes for powders, pills, syrups, decoctions, ointments, and oils. It also included recipes and instructions for preparation of theriacs–panacea-type medical concoctions as general antidotes against poisons and infectious diseases. Another pharmacopeia was compiled by Polish physician and apothecary in Krakow, **Jan Woyna** of Jasienica under the title of *Pharmacopoea Cracoviensis* and published in 1683, in compilation with two other works, *Experemintorum medico-chymicorum* and *Observationes medicochirurgico* by Daniel Matthia. This monograph was rather short; although it listed circa 1,500 apothecary ingredients in alphabetical order, the descriptions were very brief or nonexistent. Instead, multiple cross-references to other, mostly foreign pharmacopeias and dispensatoria, were provided. The only exceptions were made for new recipes developed by the author, in which case more detailed information on ingredients and methods of preparation were given. Certain new recipes were very complex, for example a novel preparation named *Spiritus Castorei Cracoviensis* consisted of four herbs, Castorei (locally sourced *Castor fiber*), Anacardini (American *Anacardium occidentale*), Asa (*Asa foetida* from Persia or Afghanistan), and Storacis (*Styrax liquidum* from Asia Minor). All four monographs, however, were private publications without any official character or certification.

In spite of the fact that unification and standardization of apothecary tradition in Europe were long overdue, the first legally enforced government pharmacopeia was published only in 1698 as *Dispensatorium Brandenburgicum*. This monograph was released in Latin language, contained descriptions of over 1,000 apothecary ingredients, including 906 complex mixtures. The most demanding recipe was that of *Theriaca Andromachi* that comprised a mixture of 71 individual components. Duke of Warsaw Frederick Augustus I made *Dispensatorium* a required apothecary regulatory document for all Polish lands in 1714 under a modified title. Following the first partition of Poland, the Carpathian foothills were divided between Poland and Austria, and two new pharmcopeias were published to regulate the apothecary practices including *Pharmacopoea Regni Poloniae* (1817) and Phаrmасороеа Austrica (1820) (Table 2). These monographs contained descriptive notes on prescribed medicines and were intended to register approved and established remedies (for the physician), and to provide formula and appropriate methods of preparation (for the pharmacist).

**Table 2:** Pharmacopeias that regulated regional apothecary practices in the sixteenth–nineteenth centuries.

**Front page**	** 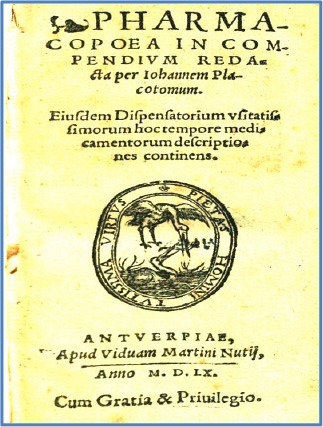 **	** 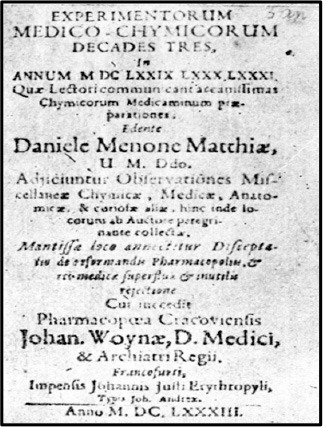 **	** 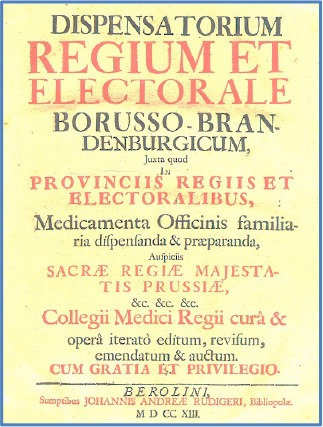 **	** 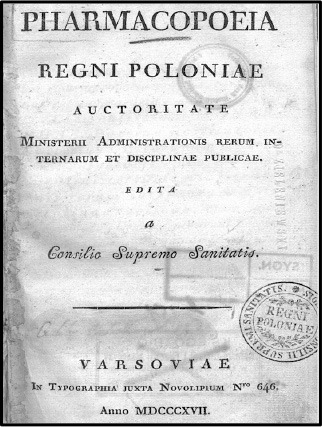 **	** 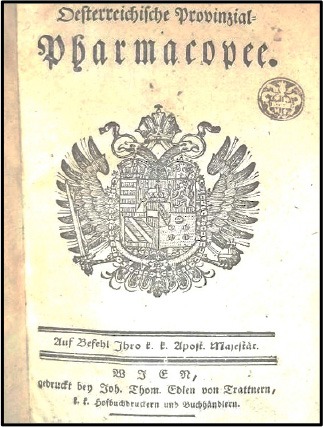 **
Author	Johann Placotomus (1514–1574)	Jan Woyna (1605–1693)	Prussian Magistrate	Polish Supreme Health Council	Austrian Pharmaceutical College
Title	*Pharmacopoea in compendium redacta per Johannem Placotomum* (Antwerp, 1560)	*Pharmacopoea Cracoviensis* (Frankfurt am Main, 1683)	Dispensatorium Regium et Electo-rale Borusso-Brandenburgicum (1714)	*Pharmacopoea Regni* Poloniae (1817)	Pharmacopoea Austrica (1820)
Notes	256 pages 502 plants total	147 pages 1,500 recipes		305 ingredients 357 mixtures	

## Handwritten herbal manuscripts from the region (sixteenth–eighteenth centuries)

The beginning of the seventeenth century marked a turning point in the use of botanicals for medicine and folk healing. The practices of trained physicians and apothecaries became chiefly summarized and regulated by pharmacopeias, while the local knowledge of healers and witchcraft practitioners was mainly restricted to oral tradition and handwritten manuscripts. While the second sources often originated from the first, they were much less consistent. These manuscripts typically borrowed from multiple printed sources, original or translated, and accreted with remarks and personal observations of the successive owners. Thus, while printed herbals recommended carline thistle (*Carlina acaulis*) to protect children from the evil enchantments of the “old women,” the same “old women” often used this plant to aid in apotropaic and love magic (Ostling, 2014).

One of the earliest examples of such manuscripts, recorded somewhere in Grand Duchy of Lithuania and self-described as “a translation from Latin and Polish books,” was created in the mid sixteenth century (designated here as **Pushkarev**). If the dating is correct, this manuscript was translated at the same time as *Hortus sanitatis* books known as “*vertograds*” were printed in Moscow (1534) and Kolomna (1588) (Novoselski and Pushkarev, 1977). The section of the manuscript that describes herbal tradition is entitled *Knigi lechebnya ot mnogih lekarev sobranye o koreniah i o zeliah* (Medicinal books collected from many doctors). Other sections contained discussions on hygiene, Galenic commentary on Hippocrates, notes and quotations from Avicenna and other medieval physicians, exerts from the pseudo-Aristotle tractates, descriptions of common medicinal practices such as bloodletting, and magical spells. Large numbers of spelling errors, incorrect interpretations and misrepresentations, all point to the fact that the manuscript was written not for personal use, but rather for sale by a professional transcriber with little knowledge on the subject. Extensive references to old Ruthenian terms *zhuravly* (cranes), *zubry* (buffalo), *tryascia* (malaria), *tsybula* (onion), *pevne* (probably), and many other words suggested Volhynia or Carpathian forelands as possible places of its origin.

The compilation of such manuscripts with no reference to the original sources was very common among Eastern Slavic groups during sixteenth–eighteenth centuries. In his *Russkije vrachebniki* work, a bibliographer of the history of Russian medicine listed 186 such manuscripts, most from the seventeenth century (Zmejev, 1895). As these manuscripts were transcribed in time, each subsequent version underwent a complex change based on the local needs and traditions, with a progressively shifting emphasis onto local and easily collectible plants, and away from their exotic and expensive alternatives. It is therefore critical in future works to establish which particular plants and beliefs were incorporated into Slavic folk healing manuscripts from European sources, which knowledge specifically reflects oral local tradition that was fixated in these works, and which part of the tradition was an original contribution of local ethnic groups. To achieve this goal, we would need to determine and investigate independent parts of handwritten herbal manuscripts (i.e., chapter groups) that originated from the common source. Another prevalent feature of these manuscripts that separated them from printed herbals was the lack of plant illustrations or prints. Otherwise, the plant uses described in these manuscripts were correct and targeted either a function of the human body (medicine), social life (relationships and feelings), household issues, or superstition beliefs (Ippolitova, 2008). With time, these descriptions became a part of the oral local tradition, and many of medicinal prescriptions and uses were eventually transferred to other unrelated plants.

A Ruthenian handwritten manuscript from the late sixteenth century, also likely a translation from a Polish source, was found in the State Archive of Bucharest (Syrku, 1883) (designated here as **Syrku**). Some of the plant descriptions from this manuscript, for example, plantain (*Plantago major*), St. Benedict's thistle (*Cnicus benedictus*), Norwegian angelica (*Archangelica officinalis*), and lesser burnet (*Pimpinella saxifraga*) were very close if not identical to other subsequent manuscripts, showing continuation of handwritten tradition in time (Nimchuk, 1976). One of them, a Ruthenian herbal manuscript *Kniha lechebnaya o mnogih lekarstv*, dated to the mid seventeenth century, survived in the Swidzinski collection from Sanok area (Peredrijenko, 1984) (designated here as **Swidzinski**). This manuscript was a compilation of various texts, legends, poems, and prayers in addition to herbal tradition. The territory to which the manuscript was localized, had formed a border line between two ethnic groups of Ruthenian origin, Boyko in the east and Lemko in the west (Falkowski and Pasznycki, 1935). Both ethnic groups still maintain a deep tradition in collecting and consuming a variety of wild plants. Among those, berries, mushrooms and hazelnuts are most popular. Several reorganizations, including the deletions of nonessential magical sections, and additions of important material about the habitat and preparation of the plant, were evident from this manuscript. We must therefore postulate, that the original knowledge about the medicinal properties of the herbs that was fixated in the earlier printed herbals, became amorphous and open to interpretation when it entered oral and written tradition of common people in the seventeenth-eighteenth centuries.

## Ethnographic studies of the carpathian foothills (nineteenth–twentieth centuries)

**Figure 2:**
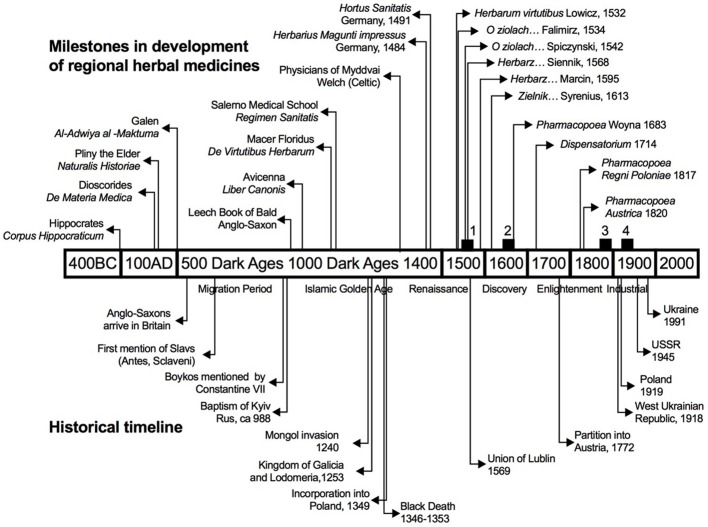
Major historical and publishing milestones that define development of regional herbal texts and botanical manuscripts. Black squares indicate the emergence of handwritten herbal mauscripts including Pushkarev (1) and Swidzinski (2), as well as ethnobotanical surveys of Rostafinski (3) and Fisher (4) discussed in this study.

While political and economic transformations took place at the lowlands of Poland and Ukraine, the people who inhabited the Carpathian ridges of the Beskid Mountains saw little change and depended mostly on cheap and readily available traditional herbal medicines for the poorest. By that time, the combined knowledge of printed herbals and handwritten manuscripts was deeply rooted in the oral tradition of folk remedies (Figure 2). This fusion was so profound, that most of the ethnographic studies of the region that took place in nineteenth and early twentieth century recorded and transcribed traditional folk medicines as a part of local cultural and ethnographic “heritage.” The rise of alternative medicines in these areas could be interpreted as one of many signs of poor development and often of an economic decline of the region. The inaccessibility and sometime active rejection of medicine, the acceptance of simplistic medical notions, and the reliance on imaginary observations indicated a loss of confidence in empirical observations and a pessimistic view of the capacity of men to overcome external difficulties by reason (Prioreschi, 2000).

Early ethnographic studies in the region were general descriptive compilations of oral traditional medicines recorded by Ukrainian and Polish enthusiasts without further analysis or comparison to the previous written works on the subject. **Danylo Chomych Lepki** described Boyko women from mountain areas who treated skin disorders in babies with herbal baths that included a mixture of pasqueflower (*Pulsatilla patens*), cornflower (*Centaurea cyanus*), rue (*Ruta graveolens*) and lesser periwinkle (*Vinca minor*), as well as the use of infusions from the bark of rowan (*Sorbus aucuparia*) and mezereon (*Daphne mezereum*) in love magic (Lepkij, 1884). **Oskar Kolberg**, a Polish ethnographer, observed the traditional use of thyme (*Thymus serpyllum*), milk thistle (*Silybum marianum*), scentless chamomile (*Anthemis arvensis*), chamomile (*Matricaria chamomilla*), wormwood (*Artemisia absinthium*), galipot (*Picea alba*), a mixture of water and burned aspen (*Populus tremula*) for treatment of osteomyelitis, and a fermented mixture of oat hulls and goose feces for treatment of abscesses among the inhabitants of the Carpathian foothills (Kolberg, 1888). **Ivan Kuziw**, a Greek-Catholic priest from Boyko villages Likot and Dydiowa, noted an unusual method of making infusions of valerian roots. They were placed in glass bottle, covered with alcohol, and baked inside a breadloaf (Kuziw, 1889). **Julian Talko-Hryncewicz** described 360 medicinal plants used in Ukrainian lowlands (Talko-Hryncewicz, 1893). **Ivan Franko**, an Ukrainian poet and ethnographer of Boyko origin, published several collections of his ethnographic observations of Boyko people, including their use of herbal medicines in magic and protection (Franko, 1898), including blessed chalk, salt, field poppy (*Papaver rhoeas*), *zillya troyan* (a mixture of *Levisticum officinale, Vinca minor*, and *Ocimum basilicum*), and garlic mustard (*Alliaria petiolata*).

**Volodymyr Szuchewycz**, an Ukrainian ethnographer and teacher residing in Lviv, observed extensive use of traditional herbal medicines by Hutsuls (a neighboring ethnic group of Ruthenian origin to the east of Boykos) and described some of the remedies in the 5th volume of his monograph (Szuchewycz, 1908), including the widely used comfrey (*Symphytum officinale*), yarrow (*Achillea millefolium*), marsh woundwort (*Stachys palustris*), mistletoe (*Viscum album*), and yellow gentian (*Gentiana lutea*). **Josef Schneider**, a Polish ethnographer born in Stebnyk, documented the use of herbal remedies by Boyko people from the Dolyna region (Schneider, 1912). He noted use of bath prepared with aspen (*Populus tremula*) and periwinkle (*Vinca minor*) for treatment of various skin disorders, baked onion (*Allium cepa*) for alleviation of abscess, green cones from spruce (*Picea abies*) for syphilis, flower petals for leg pain, and decoction of beet (*Beta vulgari*s) leaves for ringworm. **Jan Falkowski**, a Polish ethnographer, described treatment of the Polish plait (*plica polonica*, irreversibly entangled moist damaged hair) with *chorne zillya* (*Pulsatilla vulgaris*). Tinctures made with yellow gentian (*Gentiana lutea*) were a popular remedy against cholera. Special attention was paid to ashes of burned medicinal plants. Cataracts were treated by burning periwinkle (*Vinca minor*) and using the ashes to wash the eyes. Burn wounds were treated with ashes from burned wool or black ligules of sedge (*Schoenus nigricans*). Open cut wounds were treated with juice from yarrow (*Achillea millefolium*) (Falkowski and Pasznycki, 1935). Several more recent ethnographical monographs provided comparative analysis of previously published traditional knowledge and cultural differences of Boyko people, including limited data on Boyko herbal remedies. As such, they are not discussed here (Kyrchiv, 1978; Boltarovych, 1980; Hoshko, 1983). Throughout the twentieth century, there was a steady growth of published books on Ukrainian medicinal plants that also contained fragmented information on the topic from Beskids. Three of them had a particular influence on the folk knowledge of the local medicinal plants (Nosal and Nosal, 1962; Komedar, 1971; Tovstuha, 1990).

Gorale from Silesian Beskids used sap from crushed goldmoss stonecrop (*Sedum acre*) to treat ulcers. Crude, crushed herb was applied for heel pain or, if mixed with fat, into the ear to reduce earache. In Sacz Beskids it was also used internally to treat flu or urinary tract diseases. Its relaxant and emetic properties were used in food poisoning. *Sedum maximu*m leaves were applied externally crude or crushed on ulcers, bruises or edema. Sap of its leaves was mixed with barley flour and as a plaster applied to slow healing wounds (Tylkowa, 1989). On the border of Sandomierz Basin and Central Beskidian Piedmont, wound healing properties of *Sedum* were also known. It was applied in cataplasms or ointments, when fried with *Thymus serpyllum* in unsalted pork fat. Additionally, Gorale used its decoction in mouth rinsing as a gum strengthening agent or as a remedy to “destroy the scurvy impurities.” Among Gorales, several different plants had the dialect name “nine forces” but the most famous one was carline thistle (*Carlina acaulis*). The Silesian Beskids inhabitants used it both internally and externally. Its root boiled with milk or water and drank once a day on empty stomach was used in kidney and bladder disease. Leaves were put on ulcers because of its properties of getting pus out. Moreover, herb infusion was used internally to treat colic and stitching pain (Tylkowa, 1989).

**Figure 3:**
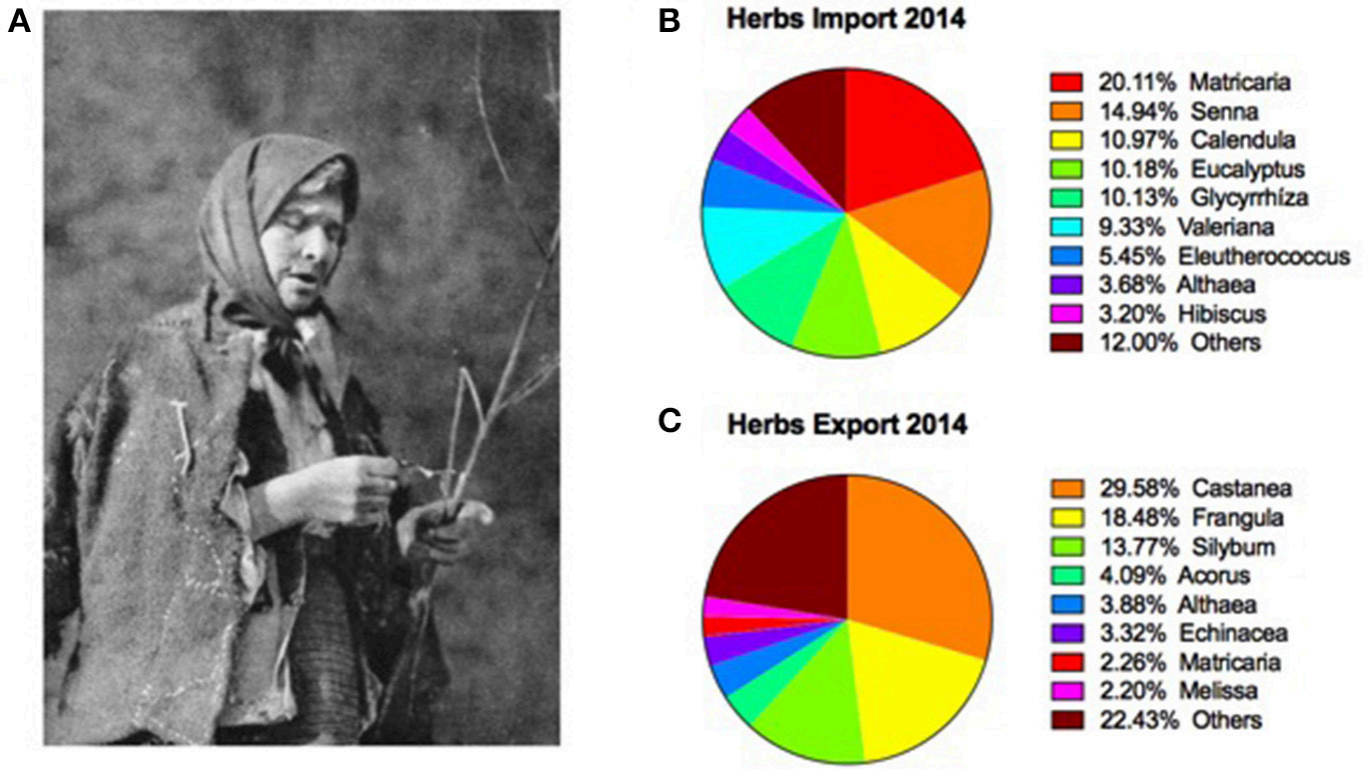
Botanical provenance of medicinal plants from the Carpathian Mountains: **(A)** Traditional healer from Eastern Carpathians as documented by Henryk Gasiorowski at the beginning of the twentieth century, and botanical profiles of major **(B)** imported and **(C)** exported medicinal plants from Ukraine in 2014 (after Nykytyuk, 2015).

It is critical to note that historical handwritten manuscripts and ethnographic studies reported equal if not larger use of verbal spells (Ua: *zagovor*, Pl: *zamawianie*), charm heals, and whispering prayers to treat diseases and cast magical incantations (Figure 3A). This knowledge was often culture and language specific, and most likely truly represented the original healing tradition that predated Roman and Greek therapies. The herbal remedies, however, were either substituted in their entirety by the knowledge gathered from translation of mostly Roman medicine, directly or via Italy and Germany, or were so similar that they could not be distinguished. This overlap though was never complete due to endemic plant species in the region, which were unknown to Greeks and Romans, and therefore could provide an exciting opportunity to observe pre-Christian folk healing traditions from the Carpathian Mountains. Guelder rose (*Viburnum opulus*) could be of particular interest in this matter as it was largely unused by the Western cultures. Similarly, the endemic species of Arran whitebeam (*Sorbus arranensis*), unknown in Roman healing tradition, was used as an antimicrobial remedy in the Scottish Highlands (Wagner et al., 2017). Previously, it was reported that the Bulgarian flora comprises 3,900 species, 12.8% of which are endemics (Petrova, 2005). Assuming a similar ratio of endemic plant species in the Ukrainian flora (6,086 species), we could expect at least 600 endemic species, of which at least 200 species could potentially contain biologically active components and be used medicinally (Konischuk et al., 2016).

## Surveys that documented traditional uses of plants in the region

Apart from the observational ethnographic studies, we are also aware of two attempts to collect and analyze ethnographical data on local use of herbal remedies in the Beskid region by mailed or published questionnaires of **Rostafinski** in 1883 (Köhler, 2015) and **Fischer** in 1929–1934 (Kujawska et al., 2015), both addressed to Polish speakers. Nearly 370 individuals replied to the first enquiry and 290 filecards were collected in the second call from the geographical region of our interest. Rostafinski's informants returned nearly 800 records of plants used by traditional societies for medical purposes. The most extensive version consisted of 70 questions concerning about 130 species. It was formulated in greater detail and asked specifically what names people used for cultivated plants, medicinal remedies (curative, ceremonial and magic plants), mushrooms, and timber. The information on the medical use of various plants obtained by Rostafinski showed that traditional homemade remedies had not yet been substituted by chemical medicines prepared by pharmacists at the time of the survey (Köhler, 2015).

Fischer's survey reported 179 plant taxa from the Galicia region. However, only 87 (49%) of them were used as medicines and targeted predominantly respiratory (24), digestive (24), and skin (23) illnesses. The remaining herbs (53 or 30%) were used for blessing during various religious ceremonies (Kujawska et al., 2015). The species that achieved the highest use were yarrow (*Achillea millefolium*), garlic (*Allium sativum*), periwinkle (*Vinca minor*), St. John's wort (*Hypericum perforatum*), and juniper (*Juniperus communis*), which was strictly in line with previous observations across various centuries. Two plants, yarrow (*Achillea millefolium*) and coltsfoot (*Tussilago farfara*) were also recognized as the most versatile remedies with multiple pharmacological indications.

## Comparative analysis of medicinal plants for wound and skin applications

To illustrate our assumption that the original Carpathian herbal remedies were either substituted by knowledge gathered from classical medicinal monographs or were identical to them, we attempted a direct comparison of descriptions of herbal medicines used to treat wounds among printed herbals and subsequent traditional folk knowledge in the region. Falimirz's herbal (1534) listed 63 plants that were used for wound healing out of 246 plants shown in Table 3 (25%). Among the herbal references analyzed, Falimirz's plant list matches 70% of plants described in the Welsh (Celtic) *Physicians of Myddvai* manuscript, even though both works are separated by a hundred years and 2,000 km–suggesting that both authors used similar, if not identical, sources for transcribing these monographs. This striking similarity could be explained in part by the fact that medieval Celtic physicians (Scottish, Irish, and Welsh) often received education at the University of Padua, similar to their Ruthenian and Polish counterparts (Scottish Historical review, 1906). Subsequent handwritten manuscripts and surveys reported both smaller numbers of total medicinal plants, as well as those used for wound healing. Only 3 plant species are recommended to treat wounds across all 6 sources studied, including *derevij* (*Achillea millefolium*), *babka* (*Plantago maior*) and *shalvia* (*Salvia officinalis*), but at least 14 plants are shared among the majority of the sources. Comfrey root (*Symphytum officinale*) was used on multiple occasions to heal wounds associated with bone fractures, minced in animal fat. The type of fat used in this ointment varied depending on the location: in Silesian Beskids, pork fat was preffered, in Zywiec Beskids, goose lard was believed to possess the best curative properties, while in Sacz Beskids they were both considered as effective. Another treatment that remained largerly uniform among the discussed regions was application of galipot of conifer trees to wound healing ointments after heating it with bee wax and butter.

**Table 3:** Medicinal plants listed in Falimirz's Herbal recipes (1534) and their occurrences in manuscripts and regional surveys, including Myddvai (M, 1382), Lowicz (L, 1532), Pushkarev (P, ~1550), Swidzinski (S, ~1650), Talko-Hrynkiewicz (T, 1893), and Fischer (F, 1934).

**Plant name**					**Appears in**					
**Old latin (Falimirz)**	***Botanical***	**English**	**Ukrainian**	**Polish (Falimirz)**	**M**	**L**	**P**	**S**	**T**	**F**
Branca ursina	*Acanthus mollis* L.	Bear's breech	Aкaнт	Barszcż, akant	x					
Platanus	*Acer pseudoplatanus* L.	Sycamore	Явiр	Jawor						
Millesolium	*Achillea millefolium* L.	Yarrow	Деревiй, рaнник, серпник	Krwawnik	x	x	x	x	x	x
Napellus	*Aconitum lycoctonum* L.	Wolfsbane	Aконiт, вовкобiй, терлич	Omieg, tojad	x				x	
Acorus, Calamus	*Acorus calamus* L.	Calamus	Aїр, лепехa, шувaр	Miecżyk, tatarak	x				x	
Capillus veneris	*Adiantum capillus-veneris* L.	Maidenhair fern	Aдiaнт венерин волос	Włoski, niekropień	x					
Agrimonia	*Agrimonia eupatoria* L.	Agrimony	Пaрило, реп'яшки, зрад, сметaнник	Rzepik	x				x	x
Gith	*Agrostemma githago* L.	Corncockle	Кукiль	Kakol	x		x		x	
Porrum	*Allium ampeloprasum* L.	Leek	Цибуля порa, порей	Luk, por	x	x	x	x	x	
Cepe, Cepa	*Allium cepa* L.	Onion	Цибуля	Cebula	x	x	x	x	x	
Allium	*Allium sativum* L.	Garlic	Чaсник	Czosnek	x	x	x	x	x	x
Althaea, Altea	*Althaea officinalis* L.	Marshmallow	Aлтея, слиз	Slaz wysoki	x	x	x		x	
Carui	*Ammi majus* L.	Bishop's weed	Aмми	Polny kmin, aminek						
Morsus galline	*Anagallis arvensis* L.	Red pimpernel	Курячi окa, мокрець	Kurzyślad, krze ziele	x				x	
Buglossa	*Anchusa azurea* P.Mill.	Anchusa	Воловик	Wołowy ięzyk		x	x			
Cinoglossa	*Anchusa officinalis* L.	Bugloss	Воловик, медуниця	Psi ięzyk, farbownik					x	
Anctum, Anethum	*Anethum graveolens* L.	Dill	Крiп	Koper	x	x	x	x	x	
Cotula setida	*Anthemis cotula* L.	Stinking chamomile	Pомaн собaчий	Rumien psi						
Cerifolium	*Anthriscus cerefolium* Hoff.	Chervil	Бугилa	Trybula	x	x	x			
Fistula pastoris	*Anthriscus sylvestris* Hoff.	Cow parsley	Бугилa	Bzdziucha, trybula	x					
Apium	*Apium graveolens* L.	Celery	Селерa	Selery	x	x				
Tela araneo	Aranea telam^*^	Spider web	Пaвутинa	Palecżyna, nic predna						
Aristologia	*Aristolochia longa* L.	Smearwort	Хвилiвник	Smolnik	x	x	x			
Aristologia	*Aristolochia rotunda* L.	Smearwort	Paсць, конопенкa, снiгур	Kokornak	x	x	x		x	
Radix	*Armoracia rusticana* G.Gart.	Horseradish	Хрiн	Chrzan			x	x		
Abrotanum	*Artemisia abrotanum* L.	Southernwood	Полин, боже деревце	Boże drzewko	x	x	x	x	x	x
Absinthcum	*Artemisia absinthium* L.	Absinthe	Полин гiркий	Piołun	x	x	x	x	x	x
Piretrum	*Artemisia dracunculus* L.	Tarragon	Тaрхун, естрaгон	Draganek, estragon	x	x		x		
Iarus	*Arum maculatum* L.	Snakeshead	Aрум	Kołorzyk, obrazki						
Asarum	*Asarum europaeum* L.	Asarabacca	Копитняк, пiдлистник	Kopytnik	x	x			x	
Sparagus	*Asparagus officinalis* L.	Asparagus	Холодок	Gromkowe korzenie	x					x
Scolopendria	*Asplenium scolopendrium* L.	Hart's tongue fern	Костенець	Jeleni ięzyk, jezycznik	x					
Atriplex	*Atriplex hortensis* L.	Saltbush, orach	Лободa, лутигa	Łoboda	x	x	x			
Berberus	*Berberis vulgaris* L.	Barberry	Бaрбaрис, кислиця, квaсниця	Berberys, piwnik	x				x	
Bleta	*Beta vulgaris* L.	Beet	Буряк	Cwikła	x				x	x
Borago	*Borago officinalis* L.	Borage	Огiрочник	Borag	x		x			x
Eruca	*Brassica eruca* Mill.	Arugula	Pуколa	Rokietta	x	x				
Caulis	*Brassica oleracea* L.	Cabbage	Кaпустa	Kapusta	x	x			x	x
Rapa	*Brassica rapa* L.	Field mustard	Piпa	Rzepa	x		x	x	x	x
Brionia	*Bryonia alba* L.	Bryony	Переступень	Przestep	x		x		x	
Herba umbilicorum	*Bupleurum rotundifolium* L.	Hare's ear	Лaскaвець	Pepownik						
Kalendula, Calendula	*Calendula officinalis* L.	Marigold	Нaгiдки, крокиш	Nogietek	x				x	x
Soldanella	*Calystegia soldanella* R.Br.	Morning glory	Плетухa	Urdzik						
Canapus	*Cannabis sativa* L.	Hemp	Конопля	Konoṕ	x		x	x	x	x
Bursa pastoris	*Capsella bursa-pastoris* Md.	Shepherd's purse	Грицики, зозульник, рiжух	Tobołki, tasznik	x				x	
Herba victorialis	*Carlina acaulis* L.	Carlina	Дев'ятисил, вiдклaсник	Dziewęsił				x	x	x
Cartamus	*Carthamus tinctorius* L.	Safflower	Сaфлор, шaфрaн	Krokosz						x
Scabiesa	*Centaurea scabiosa* L.	Knapweed	Волошкa, блевит, сонце	Dryjak, chaber, blawatek	x				x	
Centaurea	*Centaurium erythraea* Rafn.	Centaury	Золототисячник, цинторiя	Centuria, tysiącznik	x	x	x		x	x
Os de corde cervi	Cervus elaphus^*^	Red deer bone	Кiсткa оленя	Kosć z serca jeleniego						
Celidonis, Chelidonia	*Chelidonium majus* L.	Celandine	Чистотiл, глaдишник	Złotnik, glistnik, jaskolcze	x	x			x	x
Gira solis	*Chondrilla juncea* L.	Devil's grass	Хондрилa ситниковиднa	Skocżek						
Cicorea	*Cichorium intybus* L.	Chicory	Цикорiй, петровi бaтоги, стaрiвник	Podrożnik					x	
Kamphora	*Cinnamomum camphora* J.P.	Camphor laurel	Кaмфорний лaвр	Balsamowiec	x					
Calamentum	*Clinopodium nepeta* Kuntze	Lesser calamint	Кaлaмiнт	Miętka kamienna	x					
Pes corinnus	*Clinopodium vulgare* L.	Wild basil	Пaхучкa	Sturzesz, czyscica						
Hermodactilus	*Colchicum autumnale* L.	Autumn crocus	Пiзньоцвiт, зимовник	Paluchy, zimowit						
Cicuta	*Conium maculatum* L.	Hemlock	Болиголов, блекот, булaв	Swinia wesz, szczwół	x	x			x	
Lilium convalium	*Convallaria majalis* L.	Lily of the valley	Конвaлiя	Lanka, konwalia	x				x	
Coriandrum	*Coriandrum sativum* L.	Coriander	Корiaндр	Koriander, kolendra	x	x				x
Pallacium leporis	*Crepis tectorum* L.	Hawksbeard	Скердa	Pałac zaięczi, pepawa						x
Melon	*Cucumis melo* L.	Maskmelon	Диня	Melon			x		x	
Cucumer	*Cucumis sativus* L.	Cucumber	Огiрок	Ogurek	x		x		x	
Cucurbita	*Cucurbita pepo* L.	Squash	Гaрбуз	Bania, dynia						x
Amcos, Cyminum	*Cuminum cyminum* L.	Cumin	Кмин	Kmin	x	x			x	
Cuscuta, Epitimum	*Cuscuta epilinum* Weihe	Flax dodder	Повитиця, перстенець	Kania przędza, kanianka						
Panis porcinus	*Cyclamen purpurascens* Mil.	Alpine cyclamen	Циклaмен	Obraski	x					
Squinantum	*Cymbopogon schonanthus* S.	Camel grass	Цимбопогон	Palczatka						
Ciperus, Cyperus	*Cyperus longus* L.	Galilgale	Смикaвець	Cibora	x	x				
Laurcola	*Daphne mezereum* L.	February Daphne	Вовчi ягоди	Wilcże łyko, wawrzynek	x				x	
Pastinaca, Daucus	*Daucus carota* L.	Carrot	Mорквa	Marchew	x		x		x	x
Staphisagria	*Delphinium consolida* Gray	Larkspur	Дельфiнiй, сокирки	Ostróżka	x				x	
Gariosili	*Dianthus caryophyllus* L.	Carnation	Гвоздикa	Gwozdzika						x
Filius ante patrem	*Dianthus deltoides* L.	Maiden pink	Гвоздикa	Goździk						x
Virga pastoris	*Dipsacus fullonum* L.	Wild teasel	Ворсянкa, будяк, чишaки	Polna szcżeć, drapacz					x	x
Poligonia	*Dipsacus sylvestris* L.	Fuller's teasel	Ворсянкa, будяки	Szcżotki, szczeć					x	
Squilla	*Drimia maritima* (L.) Stearn	Squill	Дримiя	Czebula zamorzka						
Filix	*Dryopteris filix-mas* Schott	Fern	Щитник, пaпороть	Paproć	x					
Cauda equina	*Equisetum arvense* L.	Horsetail	Хвощ, пaдиволос	Przesika, skrzyp						x
Iringus	*Eryngium campestre* L.	Eryngo	Mиколaйчики	Mikołaiek	x				x	
Eupatorium	*Eupatorium cannabinum* L.	Hemp-agrimony	Сiдaч, дaвник, вовчки	Szadziecz	x				x	x
Esula minor	*Euphorbia esula* L.	Green spurge	Mолочaй гострий	Sosnka mniejsza						
Titimallus	*Euphorbia helioscopia* L.	Sun spurge	Mолочaй, сaмозелень	Romanowo ziele						
Catapucia	*Euphorbia lathyris* L.	Spurge	Mолочaй	Skocżek, ostromlecz	x				x	
Eufrasia	*Euphrasia rostkoviana* Hayn	Eyebright	Очaнкa	Swiecżki	x					
Stercus	Feces^*^	Feces	Кaл	Layno, vykal	x					
Elitropium	*Filipendula ulmaria* Maxim.	Meadowsweet	Гaдючник	Wiązówka	x					
Filipendula	*Filipendula vulgaris* Moen.	Dropwort	Лaбaзник, бaлaбaн	Orzeszki, wiązówka	x					
Feniculus	*Foeniculum vulgare* Mill.	Fennel	Фенхель	Włoski kopr, fenkuł	x	x				
Fragaria	*Fragaria vesca* L.	Strawberry	Суницi, ягодa	Pozimki, poziomka	x				x	
Frarimus	*Fraxinus excelsior* L.	Ash	Ясен	Jesion	x					x
Fumus terre	*Fumaria officinalis* L.	Fumitory	Pуткa, сплaвник	Dymnica	x				x	
Fungus	Fungi generaliter^*^	Mushroom	Гриб	Grzyb	x					
Camepiteos	*Galium mollugo* L.	Hedge bedstraw	Пiдмaренник, брочник	Przytulia						x
Genesta	*Genista pilosa* L.	Broom	Дрiк	Janowiec					x	
Genciana	*Gentiana punctate* L.	Spotted gentian	Тирлич, горичкa	Goricżka			x		x	x
Pes columbinus	*Geranium columbinum* L.	Longstalk cranesbill	Герaнь, кучеряве	Gołębia noga					x	
Gariossilata	*Geum urbanum* L.	Herb Bennet	Грaвiлaт, ребник, чистець, пiдоймa	Kuklik	x				x	x
Edera terrestris	*Glechoma hederacea* L.	Ground ivy	Pозхiдник, собaчa м'ятa	Bluszcz	x					
Liquiricium	*Glycyrrhiza glabra* L.	Liquorice	Локриця	Lakricija	x				x	
Gramen	*Gramen generaliter*	Hay	Ciно	Trawa	x					
Palma christi	*Gymnadenia conopsea* R.Br.	Fragrant orchid	Билинець комaрниковий	Dłoń Kristowa, golka	x					
Sticados citrinum	*Helichrysum arenarium* L.	Dwarf everlast	Цмин, жовтяниця	Koczenki, kocanki					x	
Sticados arabicum	*Helichrysum stoechas* L.	Shrubby everlast	Цмин aрaбський	Koczenki z Arabiji						
Elleborus niger	*Helleborus niger* L.	Black hellebore	Чемерник	Cżemierzycza	x	x	x		x	
Samsucus	*Hibiscus syriacus* L.	Hibiscus	Гiбiскус	Wielki slaz						
Auricula, Pilosella	*Hieracium pilosella* L.	Mousear hawkweed	Нечуйвiтер, волосник	Niedospiałek, kosmaczek	x				x	
Luppulus, Velubilis	*Humulus lupulus* L.	Hops	Хмiль	Chmiel, pawey	x					
Iusquiamus	*Hyoscyamus niger* L.	Golden henbane	Блекотa, люлян, немиця	Bielon, lulek	x	x			x	
Iperico	*Hypericum perforatum* L.	St John's wort	Звiробiй, кровкa	Jana ziele, dziurawiec	x		x		x	x
Isopus	*Hyssopus officinalis* L.	Hyssop	Гiсоп, юсипок	Izop, hyzop	x	x			x	x
Enula campana	*Inula helenium* L.	Elecampane	Омaн, дивосил, ґaлaґaн, велике	Oman	x	x	x		x	x
Ircos	*Iris germanica* L.	Iris	Пiвники	Kosaciecz						
Iris illirica	*Iris illyrica* Tomm.	Illyrian iris	Пiвники	Fijołkowy korzeń					x	
Gladiollus	*Iris pseudacorus* L.	Yellow iris	Пiвники, косaтень	Miecżyk	x		x			
Sandix	*Isatis tinctoria* L.	Woad	Вaйдa	Urzet						
Iuniperus	*Juniperus communis* L.	Juniper	Ялiвець	Jałowiecz	x		x		x	x
Savina, Sabina	*Juniperus sabina* L.	Savin juniper	Ялiвець, женепин	Sawina, jałowiec	x	x			x	
Lactuca	*Lactuca sativa* L.	Lettuce	Сaлaт	Sałata	x	x			x	
Siler montanum	*Laserpitium siler* L.	Laserwort	Pозрив-трaвa	Czarnogłow						
Lavendula	*Lavandula angustifolia* Mill.	Lavender	Лaвaндa	Lavenda	x				x	
Lenticula	*Lemna minor* L.	Duckweed	Pяскa	Rząsa	x				x	
Lens	*Lens culinaris* Medikus	Lentil	Сочевиця	Socżowicza	x					
Nasturtium ortulanum	*Lepidium sativum* L.	Garden cress	Жерухa, хрiниця	Rzeżucha, pieprzyca		x	x		x	
Oculus bouis	*Leucanthemum vulgare* Lam.	Ox-eye daisy	Королиця	Jastrun, zlocen	x				x	
Ligusticum, Leuisticus	*Levisticum officinale* Koch	Lovage	Любисток	Lubczyk	x	x	x		x	
Lilium	*Lilium candidum* L.	Madonna lily	Лiлiя бiлa	Lilija	x	x	x		x	
Assodillus, Narciscus	*Lilium martagon* L.	Martagon Lily	Лiлiя лiсовa, мaслянкa	Złotogłow, powojek						
Linaria	*Linaria vulgaris* Mill.	Toadflax	Льонок, медовики, чистець	Matki Bożey len, lnica					x	
Grana solis	*Lithospermum officinale* L.	Gromwell	Горобейник	Wroble proso, nawrot	x					x
Matrisilva	*Lonicera caprifolium* L.	Italian honeysuckle	Жимолость, козий листок	Marsylia, powoj	x				x	
Spica celtica	*Lycopodium clavatum* L.	Clubmoss	Плaвун, диреч	Widłak, babimur	x				x	x
Kantarides, Cantharides	*Lytta vesicatoria* L.	Spanish fly	Шпaнськa мушкa	Pryszczel						
Malva	*Malva sylvestris* L.	Mallow	Кaлaчики, проскурник, слизiвник	Slaz	x	x	x		x	x
Mandragora	*Mandragora officinarum* L.	Mandrakes	Maндрaгорa	Pokrzyk	x					
Epatica	*Marchantia polymorpha* L.	Liverwort	Maршaнцiя (мох)	Watrobnt ziele	x					
Maiorana	*Marrubium vulgare* L.	Horehound	Шaндрa	Szanta	x				x	
Camomilla	*Matricaria chamomilla* L.	Chamomile	Pомaшкa, ромaн, рум'янок	Rumien, rumianek	x	x			x	x
Paritaria	*Melampyrum pretense* L.	Broomrape	Перестрiч, брaтики	Nocz y dzień, pszeniec	x				x	x
Mellilotum	*Melilotus officinalis* (L.) Pall.	Melilot	Буркун	Nostrzyk, melot	x				x	
Mellisa	*Melissa officinalis* L.	Lemon balm	Mелiсa, мaточник, медовник	Miodunka, rojownik	x	x	x		x	
Piperita	*Mentha balsamea* Wild.	Peppermint	M'ятa перцевa	Pieprzycżka	x					
Menta	*Mentha spicata* L.	Spearmint	M'ятa	Miętka	x	x	x		x	x
Pulegium	*Mentha pulegium* L.	Pennyroyal	M'ятa блошинa	Mieta poley	x	x	x		x	x
Mercurialis	*Mercurialis annua* L.	Mercury	Перелiскa	Szcżyr	x		x			
Spicanardi	*Nardostachys jatamansi* DC.	Spikenard	Нaрд	Nard						
Nasturcium aquaticum	*Nasturtium officinale* Aiton.	Watercress	Нaстурцiя, жерухa	Wodna rzeżucha, rukiew	x		x		x	
Nigella	*Nigella sativa* L.	Black caraway	Чорнушкa	Kakolica, czarnuszka	x				x	x
Ungula cababellina	*Nuphar lutea* (L.) Sm.	Yellow waterlily	Глечики жовтi	Końskie kopyto, grazel						
Nenufar	*Nymphaea alba* L.	Water Lily	Лaтaття	Grzybienie	x				x	
Baselicon	*Ocimum basilicum* L.	Basil	Вaсильки, бaзил´iк	Bazylia	x				x	
Turbit	*Operculina turpethum* Mans	Turpeth	Крученi пaничi	Wilec						
Satirion	*Orchis morio et mascula* L.	Military orchid	Зозулинець	Lisie jayka, storczyk	x				x	
Diptamus	*Origanum dictamnus* L.	Cretan dittany	Maтеринкa, ясинець	Trzemdała	x		x		x	
Origanum	*Origanum vulgare* L.	Oregano	Maтеринкa, душинкa	Lebiodka, majeranek	x	x	x	x	x	x
Os mundi	*Osmunda regalis* L.	Royal fern	Осмундa	Stnisowe proso, dlugosz	x					
Pionia	*Paeonia officinalis* L.	Peony	Пiвонiя	Piwonija	x	x			x	x
Opium, papaver	*Papaver somniferum* L.	Opium poppy	Maк опiйний	Mak lekarski	x	x	x	x	x	
Oculus corvi	*Paris quadrifolia* L.	True lover's knot	Вороняче око, рaнник, бешезник	Wronie oko, czworolist					x	
Pastinaca domestica	*Pastinaca sativa* L.	Parsnip	Пaстернaк, пaстярник	Pasternak	x	x	x		x	
Serpentaria	*Persicaria bistorta* Samp.	Bistort	Гiрчaк, рaковi шийки	Węzownik, rdest	x		x	x		x
Persicaria	*Persicaria maculosa* Gray	Lady's thumb	Гiрчaк, реплик	Rdest	x		x			
Petrosilinum	*Petroselinum sativum* Fuss	Parsley	Петрушкa	Piotruszka	x		x	x	x	x
Paucedamum	*Peucedanum officinale* L.	Hog's fennel	Смовдь руськa	Wszywy kopr, gorysz	x					
Ostrus ostrucium	*Peucedanum ostruthium* Koch	Masterwort	Смовдь	Gorysz		x				
Meu	*Peucedanum palustre* Monch	Milk parsley	Горичник	Olesznik, gorysz						
Alkakenge	*Physalis alkekengi* L.	Bladder cherry	Фiзaлiс, сердечник	Michunki, zydowska wisznia					x	
Pipinella	*Pimpinella saxifraga* L.	Saxifrage	Бедринець, бедрич	Biedrzeniecz	x		x	x	x	
Pinea	*Pinus silvestris* L.	Pine	Соснa	Sosna	x				x	x
Pistacca sistica	*Pistacia terebinthus* L.	Pistacia	Терпентинне дерево	Pistacja, terebint						
Pes milui	*Plantago coronopus* L.	Miinutina	Подорожник перистий	Kania noga	x					
Amoglossa, Psilium	*Plantago maior* L.	Plantain	Бaбкa, припутник	Babka	x	x	x	x	x	x
Lingua avis	*Polygala vulgaris* L.	Tufted milkwort	Китятки	Krzyżownica						
Lingua passerina	*Polygonum aviculare* L.	Knotgrass	Спориш	Sporzysz, rdest					x	
Aaron	*Polygonum bistorta* Samp.	Bistort	Гiрчaк змiїний, рaчки, криве	Wężownik, rakowe szyjki			x		x	
Polipodium	*Polypodium vulgare* L.	Polypody	Бaгaтонiжкa, слодишкa	Paprotka	x				x	
Populus	*Populus nigra* L.	Black poplar	Тополя	Topola					x	
Portulata	*Portulaca oleracea* L.	Purslane	Портулaк	Kurza noga, portulaka	x	x			x	
Consolida, Tormentilla	*Potentilla erecta* Uspen.	Tormentil, septfoil	Перстaч, кaлгaн	Pięciornik, kurze ziele	x		x		x	x
Pentassilon	*Potentilla reptans* L.	Cinquefoil	Перстaч	Pięciornik	x					
Herba paralisis	*Primula veris* L.	Cowslip	Первоцвiт, бaрaнцi	Paraliżowe, pierwiosnek	x				x	
Simphicum	*Prunella vulgaris* L.	Self-heal	Суховершки	Sylfion, glowienka	x				x	
Persicus	*Prunus persica* (L.) Batsch	Peach	Персик	Brzoskiwia	x					x
Pulicari	*Pulicaria vulgaris* Gaertn.	Fleabane	Блошниця	Płesznik	x					
Pirula	*Pyrola minor* L.	Lesser wintergreen	Грушaнкa	Gruszyczka	x					
Lilialis	*Pyrola rotundifolia* L.	Wintergreen	Грушaнкa	Gruszyczka					x	
Galla	Galla^*^	Oak (galls)	Чорнильнi горiшки	Gallas	x		x			
Quercus	*Quercus robur* L.	Oak	Дуб	Dąb	x		x		x	
Flammula	*Ranunculus flammula* L.	Spearwort	Жовтець	Jaskier					x	
Raffanus	*Raphanus raphanistrum* L.	Radish	Pедькa	Rzodkiew	x		x		x	
Rosa	*Rosa canina* L.	Rose	Шипшинa, свербивус	Roża	x	x	x	x	x	
Rozmarinus	*Rosmarinus officinalis* L.	Rosemary	Pозмaрин	Rozmarin	x				x	
Rubea tinctorum	*Rubia tinctorum* L.	Madder	Maренa	Marzana	x				x	
Rubus	*Rubus plicatus* Weihe	Blackberry	Ожинa	Jeżyny	x				x	
Ribes	*Rubus idaeus* L.	Raspberry	Maлинa	Maliny	x				x	
Acetoja, Lapathum	*Rumex acetosa* L.	Sorrel, dock	Щaвель	Szcżaw	x	x	x		x	x
Lappacium acutum	*Rumex confertus* Wild.	Bitter dock	Щaвель кiнський	Kobyliszcżaw	x		x			
Ruta	*Ruta graveolens* L.	Rue	Pутa	Ruta	x	x	x		x	x
Salix	*Salix alba* L.	Willow	Вербa	Wirzba	x		x	x	x	
Ambrosiana	*Salvia viridis*L.	Annual clary	Шaвлiя	Szałwia	x	x				
Salvia	*Salvia officinalis* L.	Sage	Шaвлiя	Szałwija	x	x	x	x	x	x
Galletricum	*Salvia sclarea* L.	Clary sage	Шaвлiя мускaтнa	Szałwia muszkatołowa	x					
Sambucus	*Sambucus ebulus* L.	Elderberry	Бузинa, хобзa	Bez	x		x		x	
Ebulus	*Sambucus nigra* L.	Elderberry	Бузинa	Chebd, bez czarny	x					x
Sapo	*Saponaria officinalis* L.	Soapwart	Mильнянкa	Mydło, mydlnica					x	
Saturegia	*Satureja hortensis* L.	Savory	Чaбер	Cząbr	x				x	
Costus	*Saussurea costus* Lipsch.	Costus	Сосюрея	Saussurea		x				
Saxifraga	*Saxifraga granulata* L.	Saxifrage	Ломикaмiнь	Lamikamień, skalnica	x		x			
Febrifuga	*Scrophularia nodosa* L.	Figwort	Paнник, стaровинa	Trędownik	x				x	
Pellicinus	*Securigera varia* Lassen	Crownvetch	В'язiль	Wilcży groch						
Semperviva	*Sedum acre* L.	Stonecrop	Очиток, оливник	Roschodnik	x		x		x	x
Senecion	*Senecio vulgaris* L.	Groundsel	Жовтозiлля, дiдик	Przymiot, starzec	x				x	
Herba sulonum	*Serratula tinctoria* L.	Saw-wort	Серпухa	Jeleni trunk, sierpik			x			
Sinapis	*Sinapis alba* L.	Mustard	Гiрчиця	Gorcżycza	x	x	x			x
Olus	*Smyrnium olusatrum* L.	Alexanders	Смирня	Gir, przewłoka	x					
Perfeliata	*Smyrnium perfoliatum* L.	Perfoliate Alexanders	Смирня	Pepkowe ziele	x					
Solatrum	*Solanum nigrum* L.	Black nightshade	Пaслiн, нaтинник	Psianki	x					
Virga aurea	*Solidago virgaurea* L.	Goldenrod	Золотушник	Wężowe ziele, nawloc	x					
Endivia	*Sonchus oleraceus* L.	Sowthistle	Жовтий осот	Mlecż	x		x			
Spinachia	*Spinacia oleracea* L.	Spinach	Шпинaт	Szpinak						
Spongia marina	*Spongia officinalis* L.	Bath sponge	Губкa	Morzkie bdły						
Betonica	*Stachys officinalis* Trevis.	Betony	Буквиця, чистець	Bukwica	x	x	x		x	x
Premorsa	*Succisa pratensis* Moench	Devil's bit	Комонник	Cartowo ziobro, komonica	x					
Consolida maior minor	*Symphytum officinale* L.	Comfrey	Живокiст, гaвйиз	Kostywał, zywokost	x				x	x
Tenacetum	*Tanacetum vulgare* L.	Tansy	Пижмо	Wrotycż	x				x	x
Rostrum porcinum	*Taraxacum officinale* L.	Dandelion	Кульбaбa	Pepawa, mniszek	x				x	
Edera arborea	*Taxus baccata* L.	English yew	Тис, негнiй-дерево	Cis	x				x	
Serpillum	*Thymus serpyllum* L.	Thyme	Чебрець, мaтеринкa	Macierza duszka	x		x		x	x
Timus	*Thymus vulgaris* L.	Thyme	Maтеринкa	Dzięcielina, macierzanka	x					
Tribulus	*Tribulus terrestris* L.	Goats-head	Якiрцi	Oseth, buzdyganek						
Trifolium	*Trifolium pretense* L.	Red clover	Конюшинa, троян	Konik, chwast	x				x	x
Matricaria	*Triplerospermum inodorum* L.	False mayweed	Триреберник	Maruna						
Spatula fetida	*Typha latifolia* L.	Cattail	Pогiз	Pałki	x					
Urtica	*Urtica dioica* L.	Nettle	Кропивa	Pokrzywa	x	x	x	x	x	x
Usnea	*Usnea barbata* L.	Olds man beard	Уснея (лишaйник)	Mech, porost						
Moracelsi	*Vaccinium oxycoccos* L.	Cranberry	Журaвлинa	Zorawiny					x	
Valeriana	*Valeriana officinalis* L.	Valerian	Вaлер'янa, оделен	Kozłek	x				x	x
Elleborus albus	*Veratrum album* L.	White hellebore	Чемериця	Czemierzycza	x	x			x	x
Tapsus barbatus	*Verbascum thapsus* L.	Mullein	Дивинa	Dziewanna	x		x		x	x
Verbena	*Verbena officinalis* L.	Vervain	Вербенa	Koszyszcżko, werbena	x	x	x		x	
Camedreos	*Veronica chamaedrys* L.	Speedwell	Веронiкa	Przetacznik	x				x	
Viole	*Viola odorata* L.	Violet	Фiaлкa	Fijołki	x	x	x	x	x	x
Viscus	*Viscum album* L.	Mistletoe	Омелa	Jemioła	x		x			
Agnus castus	*Vitex agnus-castus* L.	Chaste tree	Витекс	Włoska wirzba	x					
Passula (uva)	*Vitis vinifera* L.	Raisins	Pодзинки	Greckie wino, rozynka	x					

* *Natural substances of non-botanical origin*.

## Modern production and use of medicinal herbs in the region

Worldwide, around 20,000 plant species are used for their medicinal properties, including 180 herbs that are recognized as medicinal by modern medicine. The annual market need for herbal and botanical raw materials is estimated at 600,000 tons, with sales reaching $7.5 billion in the United States alone, and $60 billion worlwide. Cultivation of medicinal plants remains a fast growing and sustainable section of agriculture, with profits margins exceeding those of grains and other food crops 2 to 50-fold due to development of value-addded products and parallel processing streams of raw materials and waste products. Low costs of intitial investment into growing, processing, and packaging medicinal herbs in Eastern Europe (<$ 10,000) is also a very attractive factor, however it is partially offset by lack of modern cultivation practices and delayed returns due to intial 2 to 3-year establishment of annual crop harvest (Mirzoyeva, 2013). Russia remains the largest producer of medicinal plants in the region, with Poland (cultivated herbs) and Bulgaria (wild collected herbs) in the second place. Ukrainian market of medicinal plants is rather small, with annual exports (averaging 3,000 tons) exceeding imports (averaging 2,000 tons) in most years (Nykytyuk, 2015). Botanical imports are largerly dominated by chamomile (*Matricaria chamomilla*) and senna (*Senna alexandrin*a), while chestnut (*Castanea sativa*), buckthorn (*Frangula alnus*), and milk thistle (*Silybum marianum*) are leading exports in recent years (Figures 3B,C). This is a drastic difference from 1920s, when the same region was one of the world leaders in growing, processing, and exporting medicinal plants, predominantly for German markets (Onipko, 2010). A current well-demonstrated, long-term trend toward natural medicine and consumers is indicative of increasing interest in botanical products that support whole-body health rather than focus on a specific health condition, representing a key opportunity to adequately tap the potential of agriculture- and forest-grown medicinal and aromatic plants of the Carpathian Mountains and their foreland.

## Conclusions

The first printed herbals published in the Carpathian forelands were early compilations of transcribed Greek and Roman works on the subject, and set a continuous trend of ethnic use of local botanical remedies according to their Dioscorides and Galenic qualities. The Padua-Krakow axis was identified as a key putative factor in introduction of this knowledge to Ruthenian and Polish medical students during the fifteenth–sixteenth centuries. Subsequently, this tradition was incorporated into oral and written legacy of Boyko, Lemko, and Gorale ethnic groups, and fused with Slavic use of charm and prayer heals to treat diseases and cast magical incantations. However, the traditional use of several endemic plant species in the region could provide an exciting opportunity to observe pre-Christian folk healing traditions from the Beskid Mountains. The study also highlighted the vast potential for growing and processing medicinal plants in the region, and cited low awareness among farming communities, inadequate processing capacities, price risks, and non-availability of planting material as critical constraints that need to be addressed.

## Author contributions

WK drafted ethnobiology sections of the manuscript. CW drafted and contributed knowledge on classical and Celtic medicines. EM drafted and contributed knowledge on skin and wound healing applications. AM and SK conceived, designed, and wrote the manuscript. All authors read and approved the manuscript.

### Conflict of interest statement

The authors declare that the research was conducted in the absence of any commercial or financial relationships that could be construed as a potential conflict of interest.
